# Giant Right Atrial Mass Following Surgical Aortic Valve Replacement

**DOI:** 10.5935/abc.20150066

**Published:** 2015-08

**Authors:** Teresa Bastante, Fernando Alfonso

**Affiliations:** Hospital Universitario de La Princesa, Madrid - Spain

**Keywords:** Aortic Valve/surgery, Pericardial Effusion, Heart Atria/abnormalities

A 75-year-old man underwent elective biological aortic valve replacement. Two weeks after surgery, transthoracic echocardiography (TTE) showed a small pericardial effusion. On the following day, the patient suffered from syncope. A repeated TTE revealed a "giant" echo-dense mass (90x80 mm) occupying the entire right atrium and severely limiting tricuspid valve inflow. Although the initial differential diagnosis included the development of an intracavitary process, the rapidly-growing mass with a characteristic echo‑lucent layer at its atrial aspect (consistent with the atrial wall and visceral pericardium) (arrows) led to the final diagnosis of a pericardial hematoma mimicking a huge atrial mass. Emergency surgical exploration confirmed the diagnosis.

## Figures and Tables

**Figure 1:**
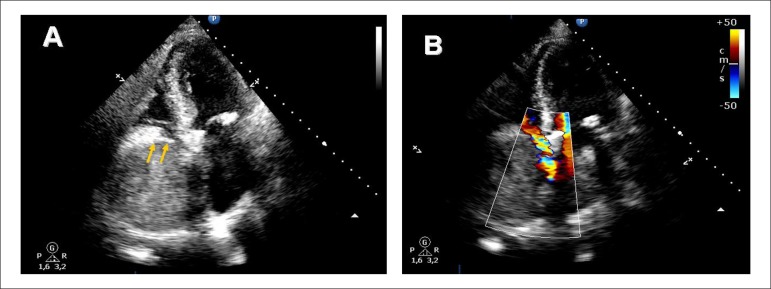
Transthoracic echocardiography: giant eco-dense mass occupying the entire right atrium (Panel A) and severely limiting tricuspid valve inflow (Panel B). Atrial wall and visceral pericardium (arrows)

